# Context-Dependence and Context-Invariance in the Neural Coding of Intentional Action

**DOI:** 10.3389/fpsyg.2018.02310

**Published:** 2018-11-27

**Authors:** David Wisniewski

**Affiliations:** Department of Experimental Psychology, Ghent University, Ghent, Belgium

**Keywords:** intentional action, volition, goal-directed action, fMRI, MVPA, prefrontal cortex, parietal cortex, context

## Abstract

Maintaining intentions over time is fundamental to goal-directed action, and previous research demonstrated that intentions are encoded and maintained in a fronto-parietal network including e.g., the dlPFC and IPS. Yet, intention maintenance is highly challenging in the constantly changing environments we experience every day. While we might have formed an intention under specific conditions, this context can change rapidly and unexpectedly. Some suggested that intentions representations in the fronto-parietal cortex change flexibly when external demands change (context-dependent coding). Others suggested that these representations are encoded in an abstract format that is not affected by changes in external demands (context-invariant coding). Here, I will first outline an analysis approach using multivariate pattern analysis of fMRI data to comprehensively assess the context-dependence / invariance of intention representations in the fronto-parietal cortex. I will then highlight some research following the proposed analysis strategy. Results to date are mixed, showing context-dependence in some, but context-invariance in other cases. In an attempt to synthesize these somewhat divergent results, I will argue that depending on characteristics of the intentions as well as the environment, intentions can either be encoded in a context-dependent or a context-invariant format. This enables us to achieve both stability and flexibility of behavior under constantly changing external demands.

## The Neural Basis of Intentional Action

Goal-directed action is central to human behavior (Shallice and Burgess, 1991; Miller and Cohen, 2001), and our ability to pursue desired goals in often volatile environments rests on intentional control of behavior (Braver, 2012; Brass et al., 2013; Koechlin, 2016). Much previous research investigated *implementation intentions* (Gollwitzer and Schaal, 1998), i.e., the intention to initiate a specific response (e.g., execute a task or action) given a specific situation (e.g., when a relevant stimulus is presented), often using a delayed-intention task (Momennejad and Haynes, 2013), and I will focus on this type of intention here as well^1^. Implementation intentions are processed in different stages, they are first formed, and then maintained over time until they can be executed (Bunge et al., 2003).

In the past, fMRI has often been used to assess the neural basis of intentional action (Jahanshahi et al., 1995; Lau et al., 2004; Zapparoli et al., 2017). For instance, activity in the medial prefrontal cortex is higher when we maintain a freely chosen intention (Forstmann et al., 2006; Brass et al., 2013). More recently, multivariate pattern analysis methods (Kriegeskorte et al., 2006; Haynes, 2015) have been used increasingly in order to identify brain regions containing information about which specific intention is currently maintained. It has been shown that the intention to perform a specific action or task is encoded in the fronto-parietal cortex, including the frontopolar (Soon et al., 2013), lateral prefrontal (Zhang et al., 2013; Muhle-Karbe et al., 2017), medial prefrontal (Wisniewski et al., 2015b), and posterior parietal cortex (Woolgar et al., 2011b; Wisniewski et al., 2015a).

These intention-related signals have been largely assessed in stationary environments that are relatively stable (e.g., repeatedly choosing between the same two tasks in the absence of any specific outcomes or experimental manipulations, Soon et al., 2013), and we currently have limited knowledge of how they might change if environments were more volatile or dynamically changing. In such environments, the fundamental problem is to ensure that intentions encoded in one specific context can still be executed if that context changes (Franklin and Frank, 2018). Here, I will tentatively use the term context to describe the immediate external and internal environment in which intentions are formed, maintained, and executed^2^. In other words, when we learn to perform a task, how do we ensure that the same task can be efficiently and reliably performed in a novel environment? Addressing this issue is demanding, not least because of methodological challenges (Bhandari et al., 2018). Yet, there have been some important attempts to assess the context-dependence of intention coding in the past, investigating e.g., the effects of reward prospect (Etzel et al., 2016) or task difficulty (Woolgar et al., 2011a)^3^. Here, I will highlight recent theoretical and methodological advances in understanding context-dependence of intention coding in the brain, and propose a comprehensive analysis strategy that will help illuminate this issue even further.

## Context-Dependent and Context-Invariant Coding of Intentions

In the past, at least two accounts have been put forward to explain how intention representations might adapt to changing external demands, emphasizing two different aspect of intention coding. First, it has been argued that the neural coding of intentions becomes more separable (or “enhanced,” for more information see Waskom et al., 2014) e.g., in contexts that make their implementation difficult (*context-dependent coding*, see, e.g., Woolgar et al., 2011a). Such flexibly changing intention coding is thought to support flexible adaptation of behavior and to ensure correct intention implementation even under difficult conditions. This argument is closely related to the adaptive-coding theory (Duncan, 2010; Fedorenko et al., 2013), which posits that some regions of the frontal and parietal cortex are part of a “multiple demand network,” a set of domain- and process-general cortical regions. This allows these regions to adapt their coding properties to cope with a wide variety of different demands, which supports flexible adaptation of behavior to changing environments. The multiple demand network partly overlaps with the network found to encode intentions, and it thus might be that contextual changes lead to changes in the coding of intentions as well. The same specific intention (e.g., brew tea) would then be encoded differently in different contexts (e.g., using a teabag vs. Japanese tea ceremony).

Second, it has been argued that intention representations might be encoded in an abstract form, which remains stable even if external demands change (*context-invariant coding*, e.g., Loose et al., 2017). This would allow the same representation to be re-used in a number of different contexts, and would thus support behavioral stability and generalization to novel environments^4^. This argument is closely related to compositional coding (Reverberi et al., 2012; Franklin and Frank, 2018), which is thought to be one of the fundamental principles underlying human learning (Kriete et al., 2013). Intentions might be encoded in a compositional format, i.e., representations of a specific intention (e.g., brew tea) would be built out of its components parts (e.g., pour water into cup + add tea). These component parts could then be re-used in different contexts (e.g., pour water into cup + add coffee). Re-using the same representations in different contexts (sometimes also called “multiplexing” Botvinick and Cohen, 2014; Naud and Sprekeler, 2018) is thought to be a highly efficient way of encoding information (but also inherently leads to issues with cross-talk between different representations, Feng et al., 2014). Given that we can never fully anticipate the specific conditions under which we will need to implement our intentions, context-invariant coding ensures we can perform under the widest range of conditions. Overall, both theories try to explain our ability to adapt our (intentional) actions under changing conditions, and emphasize different coding properties that might support this ability.

## Testing Predictions Using MVPA on fMRI Data

In a typical experiment on neural coding of intentional action, subjects are asked to encode one of two different intentions (e.g., perform an addition or subtraction task), and then need to maintain this information over a short delay period, after which they are able to execute their intention. In order to identify brain regions encoding intentions during the maintenance period, fMRI and multivariate pattern analysis (MVPA, Kriegeskorte et al., 2006; Haynes, 2015) have been used in the past (for a review see Woolgar et al., 2016).

**FIGURE 1:**
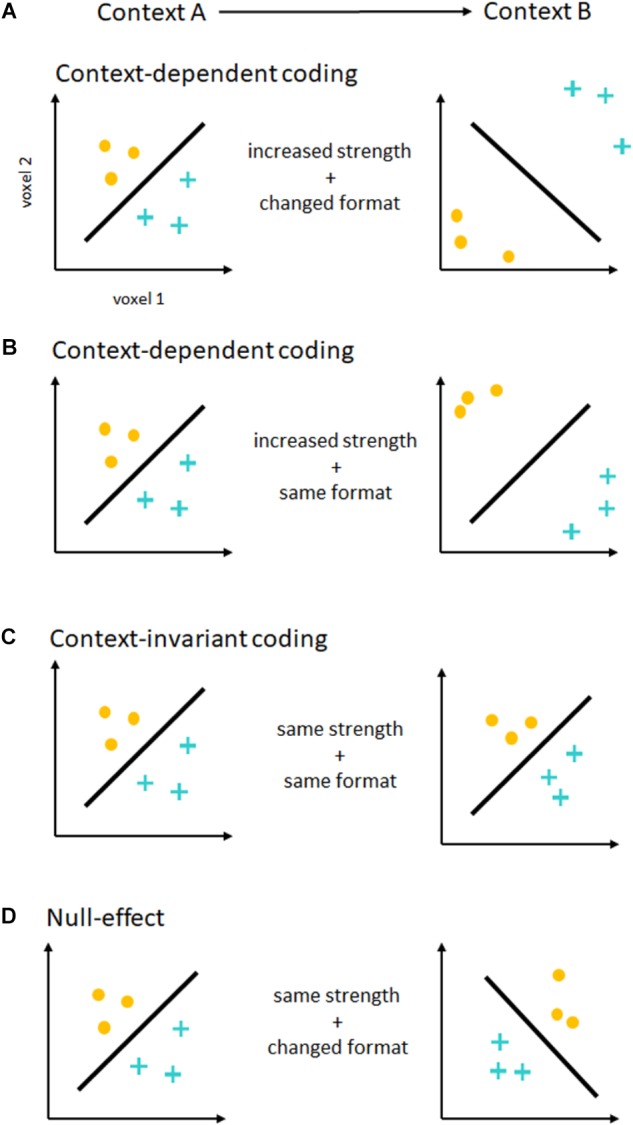
Contextual effects on the coding of intentions. Depicted are the results of a hypothetical MVPA, decoding two different intentions (yellow dots vs. blue crosses). For illustration purposes, each axis represents the activity in one single voxel. Each dot or cross represents one measurement, and the black line represents the fitted hyperplane separating both intention representations. On the left, you see decoding in one context (Context A), on the right in another context (Context B). **(A)** Context-dependent coding. If the strength of intention coding (shown as distance from the hyperplane) increases from one context to another, this would show that intentions are encoded in a context-dependent form. In this case, decoding accuracies would be higher in one than in the other context. This is true even if the representational format changes between contexts (shown as hyperplane orientation). In this example, the same hyperplane cannot be used to successfully classify different intentions in both contexts, and a cross-classification analysis would fail. **(B)** Another form of context-dependent coding. Here, the strength of intention coding increases from one context to the other, but the representational format stays similar. This would again show context-dependent coding of intentions, by means of a gain increase or amplification of intention-related signals. **(C)** Context-invariant coding. The strength of intention coding is the same in both contexts, as is the representational format. Here, there would be no difference in decoding accuracies, but cross-classification would be successful. This results pattern would be expected if intentions were encoded in a context-invariant format. **(D)** Null-effect. If there is neither a significant difference in accuracies, and cross-classification failed, results would be difficult to interpret as both analyses would show null-effects. In this case, a lack of statistical power is difficult to exclude using frequentist statistics.

In principle, contextual changes (e.g., easy vs. difficult additions) can modulate these intention representations independently along two different dimensions (Figure 1). The *strength* of intention coding, i.e., the separability or distance of neural activation patterns, can be increased from one context to the other. In MVPA, this is generally measured as an increase in decoding accuracies. For support vector classification (Chang and Lin, 2011), one of the most commonly used classification algorithms, this can reflect a greater distance of neural activity patterns from the classifier decision boundary (Figure 1B). This distance can serve as an alternative measure for the strength of intention coding (see Etzel et al., 2016). In the past, contextual effects have been shown both on decoding accuracies (e.g., Qiao et al., 2017), as well as distance to the decision boundary (Etzel et al., 2016). If there were no contextual effects on coding strength, no such differences would be expected. Assessing the strength of intention coding only shows a partial picture of contextual effects, however. To fully understand such effects on intention coding, we also need to assess the *format* of intention coding, i.e., the geometry of neural activation patterns (Kriegeskorte and Kievit, 2013), and how it changes across contexts (Figures 1A,D). In the MVPA framework, differences and similarities in coding formats across conditions can be tested using a cross-classification approach (Kaplan et al., 2015). Here, a classifier is trained in one context (e.g., easy addition vs. subtraction), and is tested in a different context (e.g., difficult addition vs. subtraction). If the representational format remains similar across contexts, significant, above chance accuracy values will be expected^5^. If the representational format changes, cross-classification will be unsuccessful. Please note that cross-classification only shows context-invariance, which is necessary but not sufficient to show compositional coding (Reverberi et al., 2012). Unfortunately, despite MVPA’s potential to separately assess effects on intention coding strength and format, this important distinction has not been made explicitly in much of the past research.

If we are to draw strong conclusions about the context-dependence of intention coding from an experiment employing MVPA, we need to perform both a comparison of decoding accuracies across contexts, as well as a cross-classification across contexts. Context-dependent coding would predict stronger coding of intentions in one context than the other (Woolgar et al., 2011a; Etzel et al., 2016; Qiao et al., 2017). Although context-dependent coding seems to suggest changes to coding formats across contexts as well, no strong claims in this direction have been made in the past, so that this account does not seem to have a strong position on intention coding formats. Context-invariant coding on the other hand would predict no contextual effects on coding strength, and would predict similar coding formats in both contexts (see Zhang et al., 2013; Wisniewski et al., 2016). Please note that this does not constitute a case of arguing the null, as this results pattern requires significant findings in a cross-classification analysis (see also Wisniewski et al., 2018).

## Previous Evidence

Having outlined an analysis approach that allows us to test comprehensively whether intentions are encoded in a context-dependent or context-invariant way, we can now turn to previous evidence on this issue. Unfortunately, most of the previous studies (Table 1) focused only on effects on coding strength and did not directly assess effects on representational formats (Woolgar et al., 2011a; Hebart et al., 2012; Nee and Brown, 2012; Waskom et al., 2014; Etzel et al., 2016), and conclusions from these findings remain limited. Some previous research did follow the analysis strategy outlined above, however (Momennejad and Haynes, 2013; Zhang et al., 2013; Wisniewski et al., 2016, 2018; Loose et al., 2017; Qiao et al., 2017), and we will focus on these findings here.

**Table 1:** Previous studies investigating context-effects on intention coding.

Name	Context manipulation	Coding strength	Coding format	n
		Difference in coding strength?	Invariant coding?	
Etzel et al., 2016	reward vs. no reward	yes	not tested	20
Hebart et al., 2012	easy vs. difficult task	yes	not tested	22
Loose et al., 2017	task switch vs. repeat	no	yes	38
Momennejad and Haynes, 2013^+^	high vs. low cognitive load	yes	yes	23
Nee and Brown, 2012	abstract vs. concrete rules	yes	not tested	21
Qiao et al., 2017	task switch vs. repeat	yes	no	44
Waskom et al., 2014	task switch vs. repeat	yes	not tested	15
Wisniewski et al., 2016	free vs. cued intentions	no	yes	31
Wisniewski et al., 2018	contingent vs. non-contingent rewards	no	yes	35
Woolgar et al., 2011a	easy vs. difficult task	yes	not tested	18
Zhang et al., 2013^+^	free vs. cued intentions	yes	yes	19


*For each study, the context manipulation used and sample size (n) is shown. Furthermore, it is shown whether effects on coding strength and/or coding format were found, or whether this was not assessed. This list is not exhaustive and only represents the research focused on the most in this article. ^+^found context-dependent coding in some, and context-invariant coding in other brain regions.*

In these previous papers, four different contextual variables were assessed. Two experiments assessed the effect of freely choosing an intention vs. being externally cued which intention to choose (Zhang et al., 2013; Wisniewski et al., 2016). Both experiments demonstrated context-invariant coding in the parietal cortex, with less consistent results in the prefrontal cortex. While Zhang and colleagues showed context-dependence in the lateral prefrontal cortex, Wisniewski and colleagues showed context-invariance. Other experiments assessed the effects of high vs. low cognitive control demands on intentions coding in a task switching paradigm (Loose et al., 2017; Qiao et al., 2017). Again, results are not consistent, with one study showing context-invariant coding in frontal and parietal brain regions (Loose et al., 2017), and another study showing context-dependent coding in these brain regions (Qiao et al., 2017). One study assessed the effect of high vs. low cognitive load (Momennejad and Haynes, 2013), and found that some brain regions showed context-invariant coding (pre-SMA, lateral frontopolar cortex), while others showed context-dependent coding (vmPFC, posterior temporal cortex). The last study assessed the effect of choice-contingent vs. non-contingent reward outcomes, and found context-invariant coding in the parietal and lateral prefrontal cortex (Wisniewski et al., 2018).

Overall, results seem somewhat inconsistent. Given the small number of studies performed to date, this is not entirely surprising, and more evidence is clearly needed in order to draw robust conclusions. One possible explanation could be that these studies show substantial differences in their experimental designs, which could lead to increased variance in the results. It has been pointed out that, e.g., contrasting different cognitive tasks (e.g., adding vs. subtracting) vs. contrasting different stimulus-response (SR) mappings (e.g., odd number → left button vs. odd number → right button) might explain at least some of the observed differences (Qiao et al., 2017). Indeed, two SR mappings will often be more similar to each other than two cognitive tasks. Using SR mappings might thus bias results toward similar / context-invariant coding (which makes context-dependent coding for SR mappings a stronger result, see Woolgar et al., 2011a). Conversely, using cognitive tasks might potentially bias results toward dis-similar/ context-dependent coding (which makes context-invariant coding for different tasks a stronger result, see Wisniewski et al., 2016).

## Context-Dependence and Invariance Are Not Mutually Exclusive

Given the previous evidence, it seems unlikely that intentions are encoded solely in a context-dependent or context-invariant form. These two views are not mutually exclusive, and using both encoding formats has some potential benefits. In the case of visual perception, it has been argued that the brain has both context-dependent and context-invariant processing systems, which are anatomically separated (Xu, 2018). This helps maintaining stable representations of visual objects while also being able to flexibly react to changing environments. A similar division of coding formats might be present for intention coding as well, although an anatomical separation is less likely in this case (but see Momennejad and Haynes, 2013). A more likely scenario is that the fronto-parietal network can exhibit both context-dependent and context-invariant intention coding.

Of course, in this case one or the other encoding format needs to be selected. This will likely depend on which format is more useful in reaching desired goals, and both context-dependent and context-invariant coding have associated costs and benefits. Context-dependent coding allows us to flexibly react to rapidly changing demands, at the cost of potentially limited generalization to novel conditions (Botvinick et al., 2009). Context-invariant coding allows us to easily generalize existing intention representations to novel conditions, but at the cost of increased cross-talk between different representations (Feng et al., 2014). One thus might predict e.g., that intentions implemented in frequently changing and novel contexts should be encoded in a context-invariant form, and this prediction should be tested directly in future research. The structure of the environment is also key in determining the format of intention coding. First, the degree to which contextual changes are behaviorally relevant likely plays a role. It seems that contextual manipulations that strongly affect the implementation or performance of the chosen intention (e.g., difficulty, reward prospect) lead to more context-dependent coding (Woolgar et al., 2011a; Etzel et al., 2016), while manipulations that have subtler behavioral effects (e.g., free vs. cued intentions) lead to more context-invariant coding (Zhang et al., 2013; Wisniewski et al., 2016). Second, the similarity of different contexts likely plays a role as well. Sometimes, different contexts will be similar, allowing us to implement our intentions in a relatively similar fashion (e.g., brewing green tea vs. black tea). At other times, different contexts will be highly dis-similar, and our intentions will need to be implemented in very different ways (e.g., brewing tea using a teabag vs. Japanese tea ceremony). Recent evidence from computational modeling suggests that this similarity between contexts plays a large role in how we generalize behavior from one context to the other (Franklin and Frank, 2018). If contexts are similar, and we can implement our intentions in largely the same way, we should see more compositional or context-invariant coding (Reverberi et al., 2012), as this facilitates generalization across such contexts. Overall, it seems likely that intentions can be encoded both in a context-dependent and context-invariant format, and that both characteristics of the intentions themselves as well as the environment will determine which format will be more useful to reach desired goals.

## Future Directions

In the previous sections, I described two accounts of how intentions can be encoded in changing environments: context-dependent vs. context-invariant coding. Using MVPA, we are able to assess contextual effects both on the strength and format of intention coding in the fronto-parietal cortex, although this important distinction has not been made explicitly in much of the previous research. Importantly, by investigating effects on both, we can assess evidence for and against context-dependent and -invariant coding, and determine which coding format is used under which circumstances. Evidence for both types of intention coding have been found in the past, and the specific type of intention coding likely depends on both characteristics of the intentions as well as environments.

Clearly, there still remain a number of both empirical and theoretical questions. Here, I highlighted the usefulness of MVPA methods to address context-dependence of intention coding. Representational similarity analysis (Kriegeskorte, 2011) might offer an alternative analysis approach that is able to address similar issues, and in the future we might see more experiments using this method (see e.g., Qiao et al., 2017). Furthermore, we will need to address much more systematically under which specific conditions the brain encodes intentions in a context-dependent or -invariant way. Above, I have shown that both the similarity of different contexts, as well as their effects on behavior likely play a key role. What is currently missing is a more precise definition and a “taxonomy” of different contexts, i.e., a system to define and classify contextual manipulations into different types. For instance, one possible dimension along which contextual manipulations could be organized is “external” vs. “internal.” More external contextual changes like reward outcomes (Etzel et al., 2016) might lead to different effects on intention coding strength / format than more internal contextual changes like cognitive load (Momennejad and Haynes, 2013), possibly mediated by differential effects on behavioral performance. A systematic assessment of different types of contextual changes will help us determine boundary conditions for context-dependent and -invariant coding in the future. Addressing these issues will help us understand how we are able to implement our intentions consistently, yet being able to react flexibly to the constantly changing environments we are faced with every day.

## Author Contributions

DW designed this research and wrote the article.

## Conflict of Interest Statement

The author declares that the research was conducted in the absence of any commercial or financial relationships that could be construed as a potential conflict of interest.

## Footnotes

1 Implementation intentions are closely related to concepts like task-set (Sakai, 2008), proactive control (Braver, 2012), or prospective memory (McDaniel and Einstein, 2000). I will use the term (implementation) intention to broadly describe these and related processes.

2 There is currently no consensus on a precise definition of “context” in the literature on intentional action, and the proposed definition remains tentative. This is likely because the “environment” can be described along many different dimensions. Some of these are more related to external factors, e.g., motivational (different reward outcomes (Etzel et al., 2016)), or spatial contexts (breakfast table vs. supermarket Uithol et al., 2018). Other factors are more strongly related to internal, or task-related factors. For instance, intending to perform a difficult task (e.g., solving an equation in a final math exam) is different to intending to perform an easy task (e.g., splitting a restaurant bill in half, see also Woolgar et al., 2011a), likely due to differences in anticipated effort. Also, freely choosing a task is different from being told which task to perform (Wisniewski et al., 2016). The unifying feature of all these examples is that some (external or internal) environmental variable likely affects the formation, maintenance, or execution of intentions in the brain.

3 The issue of context-dependence is also being discussed in the literature on prospective memory (McDaniel and Einstein, 2000). Given that prospective memory and intentional action are closely related concepts, the interested reader is pointed to e.g., (Bugg and Ball, 2017; Pedale et al., 2017) for more details. The method proposed here to assess context-dependence of intention coding is also applicable to prospective memory research, and might be of use there as well.

4 Here, I focus on effects of different specific contexts or environments. For a discussion of flexible coding across time (within the same context) see e.g., (Sigala et al., 2008; Stokes et al., 2013; Wisniewski et al., 2015a).

5 Please note that there are alternative methods to assess the format of intention coding in the brain, e.g., representational similarity analysis (Kriegeskorte, 2011), or repetition suppression (Barron et al., 2016). However, given that most previous research on this issue used multivariate classification I will focus on this approach here.
